# Glioblastoma as a neuro-immune network disorder: rethinking the tumor microenvironment, neural circuit integration, and therapeutic resistance

**DOI:** 10.3389/or.2026.1906725

**Published:** 2026-07-27

**Authors:** Tyler Lo, Armando Bunjaj, Jeffrey P. Turnbull, Shivum Desai, Ammar Alsalahi, Heather Heitkotter, Daniel W. Griepp, Boyd F. Richards

**Affiliations:** 1 Division of Neurosurgery, Henry Ford Health System Providence Hospital, College of Human Medicine, Michigan State University, Southfield, MI, United States; 2 Department of Medical Education, Touro College of Osteopathic Medicine, Great Falls, MT, United States; 3 Department of Medical Education, Lake Erie College of Osteopathic Medicine, Elmira, NY, United States

**Keywords:** CNS WHO grade 4, high-grade glioma, IDH-wildtype glioblastoma, immunotherapy, neuro-immune network, tumor microenvironment

## Abstract

Glioblastoma, IDH-wildtype, CNS WHO grade 4, is a highly aggressive primary tumor of the central nervous system characterized by infiltrative growth, marked antigenic heterogeneity, and resistance to treatment. Despite advances in immunotherapy, clinical responses of glioblastoma remain transient and non-durable. Emerging evidence suggests that glioblastomas and related high-grade gliomas reside within a highly regulated neuro-immunologic tumor microenvironment (TME), which may contribute to these limitations. Within this microenvironment, structural, biochemical, and cellular remodeling reduce the efficacy of current immunotherapy, including chimeric antigen receptor (CAR) T-cell therapy and immune checkpoint inhibitors, by impairing lymphocytic infiltration across the blood-brain barrier (BBB) and promoting T-cell exhaustion. The refractory nature of these tumors is further influenced by the neural circuitry that surrounds the TME. Through signaling molecules, such as glutamate and neuroligin-3 (NLGN3), neuronal activity can predispose the TME to an immunosuppressive baseline while simultaneously advancing tumor cell proliferation. These upstream signaling pathways and regionally heterogeneous neural interactions may contribute to diverse immune phenotypes and behaviors that ultimately influence clinical outcomes. These findings support a shift from a tumor-centered view to a neuro-immunological network model. Future therapeutic strategies will likely require a multidisciplinary approach that integrates neural signaling pathways, immune system modulation, and spatially defined landscapes, thereby reframing glioblastoma and related high-grade gliomas as a systems-level disorder rather than an isolated malignancy.

## 1 Introduction

Glioblastoma, IDH-wildtype, CNS WHO grade 4 is the most aggressive primary malignancy of the central nervous system, characterized by its rapid proliferation, antigenic heterogeneity, and resistance to therapy (1–4). Histologically, glioblastoma demonstrates microvascular proliferation, pseudopalisading necrosis, and high mitotic activity (1). Molecularly, these tumors are defined by IDH-wildtype status and commonly demonstrate alterations involving the TERT promoter, EGFR amplification, or combined whole-chromosome seven gain and whole-chromosome 10 loss, molecular features that independently support a diagnosis of glioblastoma (3–5). In contrast, astrocytoma, IDH-mutant is graded within a distinct molecularly defined tumor type and may be assigned CNS WHO grade 4 status based on the presence of microvascular proliferation, necrosis, or homozygous deletion of CDKN2A/2B (3–5). These distinctions are clinically important, as IDH status is strongly associated with tumor prognosis and therapeutic outcome (3,4,6).

The current standard-of-care treatment consists of maximal surgical resection followed by radiotherapy with concurrent temozolomide (7). While this approach improves survival, prognosis remains poor, with a median overall survival of 15–21 months and 5-year survival rates between 5% and 13% (8,9). Recent immunotherapeutic strategies, including chimeric antigen receptor (CAR) T-cell therapy and immune checkpoint inhibitors (ICIs), have demonstrated preliminary promise but have yet to yield durable improvements in long-term outcomes (10).

These therapeutic limitations suggest that prevailing models of glioblastoma pathophysiology incompletely capture factors that drive tumor progression and therapeutic resistance. Traditional frameworks often conceptualize glioblastoma as a tumor-intrinsic process and therefore emphasize cytotoxic immune responses directed primarily against malignant cells. However, emerging evidence indicates that glioblastoma and other high-grade gliomas are shaped by a broader microenvironment where immune regulation, stromal remodeling, vascular dysfunction, and neuronal signaling interact through continuous bidirectional communication (11–21). This review proposes that these tumors may be more accurately understood as a neuro-immune network disorder in which immune suppression, synaptic integration, and spatially defined microenvironments collectively drive tumor invasion, immune evasion, and therapeutic resistance. We further discuss how this framework may influence neurosurgical intervention and perioperative therapeutic design, with implications for future immunomodulatory approaches to glioblastoma and related high-grade gliomas.

## 2 The immunosuppressive architecture of the tumor microenvironment

### 2.1 Structural remodeling: the extracellular matrix and the blood-tumor barrier

The tumor microenvironment (TME) constitutes a multilayered system composed of structural, metabolic, and cellular elements that promote tumor progression while simultaneously suppressing antitumor immune responses (22). Much of this literature has been established in glioblastoma models, but several mechanisms, including extracellular matrix (ECM) remodeling, glioma stem-like cell signaling, and hypoxia-driven immunosuppression, have additionally been described in broader high-grade glioma contexts (22–29). Within this system, the neural ECM undergoes extensive remodeling, contributing to immune dysregulation, tumor recurrence, and therapeutic resistance (23).

One mechanism of ECM remodeling is mediated by mesenchymal stem cells (MSCs), which secrete metalloproteinases that degrade ECM components and facilitate tumor invasion (30–32). MSCs additionally interact with glioma stem-like cells (GSCs) to activate the IL-6/STAT3 signaling axis, which promotes angiogenesis and reinforces a pro-tumorigenic state (31,33). Sustained activation of this pathway increases myeloid cells’ expression of PD-L1, leading to regulatory T-cell expansion and suppression of effector T-cell activity (34).

In addition to matrix degradation, glioblastoma-associated stromal interactions further contribute to ECM reorganization by altering matrix stiffness. Normal neural parenchyma is typically characterized by low collagen deposits and high compliance, whereas glioma-associated remodeling may further increase ECM stiffness through signaling pathways that promote lysyl oxidase secretion, driving collagen cross-linking, matrix rigidity, and subsequent tumor infiltration (32,35,36). These observations suggest that ECM remodeling actively shapes immune trafficking and accessibility within the TME rather than functioning solely as a structural scaffold.

Structural remodeling is directly linked to vascular dysfunction within the TME. In glioblastoma and other brain tumors, the blood-brain barrier is reconstituted as a nonuniform blood-tumor barrier (BTB) characterized by abnormal vascular permeability and transport function (37,38). These alterations increase interstitial fluid pressure, reduce tumor perfusion, and elevate mechanical stress, which stimulates vascular endothelial growth factor (VEGF) secretion (39). In addition to promoting tumor angiogenesis, VEGF suppresses dendritic cell function and the expression of intercellular adhesion molecule-1 and vascular cell adhesion molecule-1, thereby impairing T-cell infiltration and further reinforcing hypoxic conditions within the TME (22,40).

### 2.2 Metabolic and cellular drivers of immunosuppression

Hypoxia reshapes the immune function of astrocytes, GSCs, and other immune cells, including tumor-associated macrophages (TAMs) (24–29). Under oxygen-limited conditions, astrocytes undergo HIF-2α-mediated reactive changes accompanied by the secretion of IL-3, TGF-β1, angiogenin, and VEGF-A, further enabling tumor proliferation and vascular remodeling (24).

During periods of metabolic stress, GSCs exhibit adaptive metabolic programming and protective autophagy, which help maintain stem-like properties while recycling intracellular components to sustain cellular survival (25,26). Hypoxia additionally promotes a shift towards anaerobic glycolysis, which increases resistance to temozolomide and supports tumor progression in nutrient-deprived regions (27).

The immune populations within the TME are similarly affected. Reduced oxygen availability facilitates TAM recruitment and favors M2-like polarization, characterized by TGF-β and IL-10 secretion, which ultimately limits CAR T-cell therapeutic responses (27–29). Hypoxia also impairs antigen presentation by downregulating major histocompatibility complex (MHC) class II expression on dendritic cells (29). These metabolic alterations establish a microenvironment inherently resistant to immunotherapeutic intervention, even before treatment initiation.

## 3 Limitations of immunotherapy in glioblastoma

Immunotherapy has emerged as a promising therapeutic strategy for glioblastoma with current approaches primarily centered on ICIs and CAR T-cell therapy (10,41). CAR T-cell therapy involves the genetic engineering of T lymphocytes to recognize tumor-associated antigens, independently of MHC-restricted antigen presentation (42). Recent clinical studies have investigated several glioblastoma-associated antigens, including epidermal growth factor receptor variant III, human epidermal growth factor receptor 2, and interleukin-13 receptor alpha 2, with initial trials demonstrating preliminary antitumor activity (10,41).

ICIs function by disrupting inhibitory signaling cascades that normally suppress T-cell activation and differentiation (43). While the most commonly studied targets in glioblastoma involve cytotoxic T-lymphocyte-associated protein four and programmed cell death protein 1 (PD-1), other checkpoint molecules, including TIM-3 and LAG-3, have additionally been implicated in glioma-associated immune responses (44).

Despite early encouraging findings, durable therapeutic responses remain limited. A major challenge is the profoundly immunosuppressive nature of the glioblastoma microenvironment (11). Structural remodeling within the TME, particularly the formation of the BTB, restricts effective drug delivery and impairs immune cell trafficking into the tumor bed (37,45). Underlying antigenic heterogeneity further limits the efficacy of CAR T-cell therapy through dynamic variation in antigen expression across diverse tumor cell populations (41). Metabolic adaptations within the TME, including hypoxia-driven signaling, additionally weaken immunotherapeutic responses by promoting regulatory immune phenotypes and suppressing antigen presentation (29).

These limitations suggest that current immunotherapeutic strategies in glioblastoma may be restricted by a broader microenvironment that actively maintains immunosuppression. More effective therapeutic approaches may therefore benefit from interventions that extend beyond malignant cells to address the structural, metabolic, vascular, and neuronal signaling pathways that shape tumor progression. Immunotherapy resistance may reflect not only impaired immune activation but also upstream regulatory processes within the local neuro-immune microenvironment that collectively sustain immunosuppression.

## 4 A neuro-immune network disorder

### 4.1 Neuro-immune network framework

**FIGURE 1:**
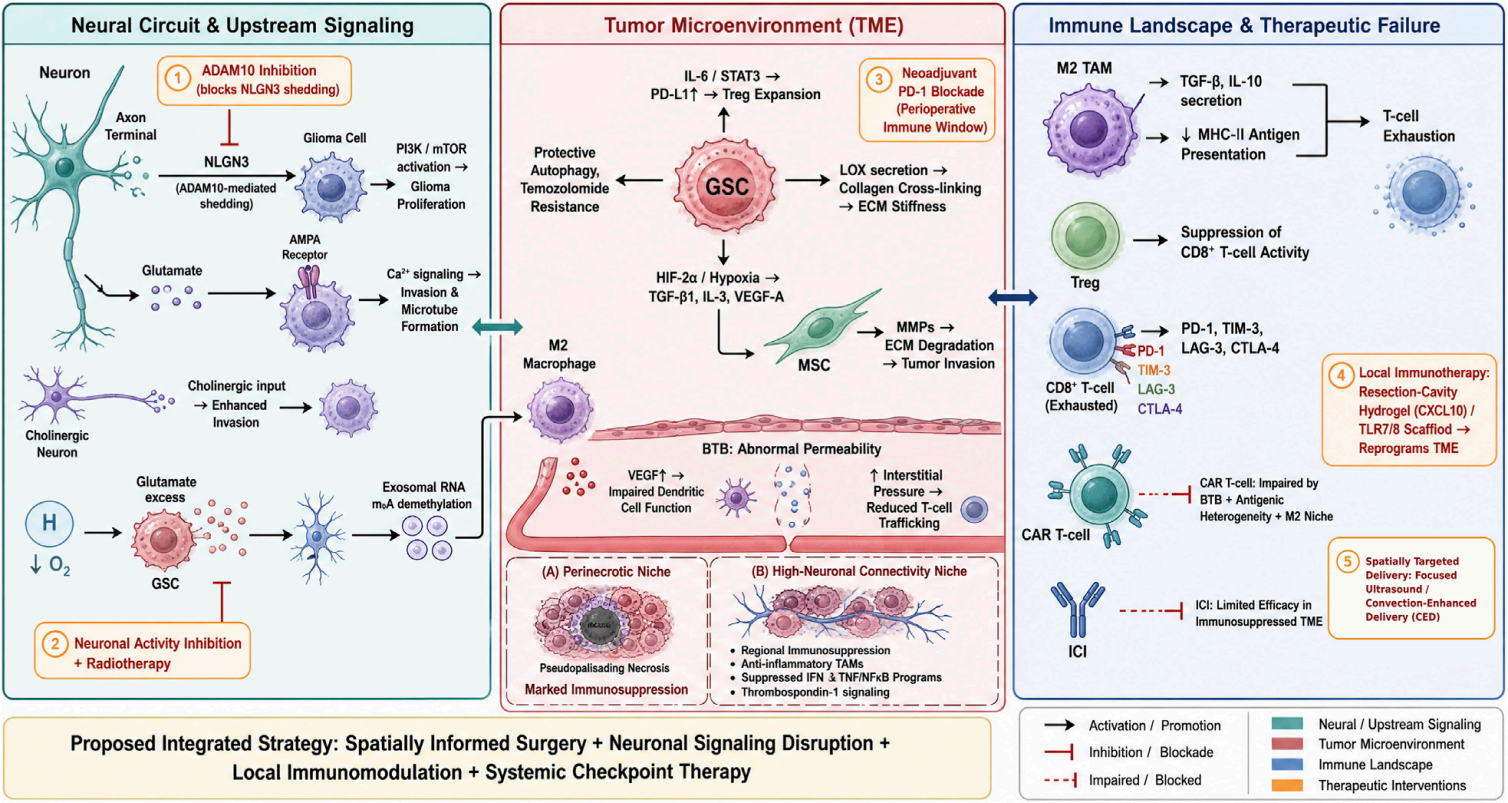
Key signaling pathways and therapeutic intervention points of the proposed neuro-immune network model of glioblastoma.

Prior reviews have discussed the immunosuppressive glioblastoma microenvironment and the emerging neuro-immune axis in gliomas (11,21). Our proposed framework differs in three important respects. First, it integrates neuronal activity, immune suppression, and spatial heterogeneity as interdependent processes operating within a systems-level network, rather than independent pathways. Second, we emphasize neuronal activity as an upstream regulator of immune phenotype—not merely a co-existing feature—supported by direct experimental evidence from human tissue and retrograde circuit tracing (19,20,46,47). Third, we propose that this network-level integration has direct implications for the sequencing and spatial targeting of surgical and immunotherapeutic interventions (Figure 1).

### 4.2 Upstream immune regulation

One of the most important advances in modern glioma biology is the recognition that neuronal activity is not merely a background feature of tumor location; rather, it is an active participant in driving disease progression (13–20) (Table 1). A major implication of the network-centric model is that neuronal activity may contribute to the establishment of immune dysfunction in high-grade gliomas rather than representing a downstream consequence of tumor growth (21). Seminal work by Venkatesh et al. in 2015 demonstrated that neurons actively promote high-grade glioma proliferation by secreting activity-dependent neuroligin-3 (NLGN3), which activates the PI3K-mTOR signaling pathway and drives feedforward NLGN3 expression in glioma cells (13). Subsequent research demonstrated that ADAM10-mediated shedding is required for this mitogenic effect, enabling NLGN3 release into the TME (14). These observations demonstrated that neuronal activity is not merely associated with glioma growth but can provide a direct trophic input into tumor progression; notably, NLGN3 expression levels in human high-grade glioma were negatively correlated with overall patient survival (13,14).

**TABLE 1:** Landmark studies supporting the emergence of the neuro-immune network of high-grade gliomas.

Study	Concept	Relevance to the neuro-immune network model
(13)	Activity-dependent neuroligin-3 identified as a high-grade glioma mitogen	Established neuronal activity as a direct driving force for glioma growth
(14)	ADAM10-dependent neuroligin-3 shedding identified as a targetable dependency in high-grade glioma	Shifted neuron-glioma biology from observation to therapeutic tractability
(15)	Glutamatergic synaptic input to glioma cells	Demonstrated direct neuron-to-glioma synaptic communication driving tumor progression
(16)	Glioblastoma hijacking neuronal mechanisms for brain invasion	Linked neuronal activity with calcium signaling, tumor microtube formation, and invasion in glioblastoma
(48)	Remote neuronal activity promotes high-grade glioma progression through SEMA4F paracrine signaling	Highlights the neuron-glioma axis extends beyond local synaptic input to include systemic circuit-level contributions
(46)	Glioblastoma functionally remodels human neural circuits, with network integration correlating with reduced patient survival	Provided clinical evidence that neuron-tumor circuit remodeling is an active determinant of patient outcome
(47)	Integrated spatial analysis revealed multi-level cellular communities in glioblastoma with co-varying malignant and non-malignant populations	Demonstrated that higher-order tissue interaction linked spatial architecture to intrinsic tumor biology
(17)	Hypoxia-driven neuronal activity promoted microglial M2 polarization and subsequent glioma progression	Provided evidence of neuronal signaling influencing immune phenotype and establishing an immunosuppressive niche
(18)	Spatial transcriptomics identified tumor cell states and marked immunosuppression within the perinecrotic niche of glioblastoma	Strengthened the spatial component of the model by showing distinct regional immune niches
(19)	Glioblastoma neuron-tumor networks were characterized with retrograde tracing	Showed that glioblastoma integrates into distributed neural circuits, linking connectivity to invasion and therapeutic resistance
(20)	Glioma-neuronal circuit remodeling directly linked to regional immunosuppression	Provided direct evidence of local neuronal connectivity shaping the local immune phenotype within glioma tissue

The biologic significance of neuron-glioma communication was further strengthened by the demonstration of glutamatergic synapses between neurons and glioma cells (15). In this work, AMPA receptor-mediated synaptic input generated postsynaptic currents in glioma cells and likely contributed to calcium-dependent invasion and tumor growth, thereby providing a direct electrophysiologic route through which neuronal activity can influence malignant behavior. More recently, Venkataramani et al. in 2022 demonstrated that glioblastoma cells can hijack nearby neuronal mechanisms to migrate into the neural parenchyma, with these signaling pathways promoting *de novo* microtube formation and enhancing the invasiveness of GSCs (16). Extending these findings, Huang-Hobbs et al. in 2023 identified that remote neuronal activity drives glioma progression through SEMA4F signaling, providing evidence that non-localized neuronal circuits contribute to tumor growth through paracrine mechanisms (48). Greenwald et al. in 2024 used integrated spatial analysis to demonstrate that glioblastoma is organized into multi-layered cellular communities in which malignant and non-malignant populations co-vary in structure and physiology, facilitating higher-order tissue interaction (47). Krishna et al. in 2023 provided supporting clinical evidence by highlighting how glioblastoma functionally remodels human neural circuits that lead to tumor progression and reduced patient survival, establishing that neuron-tumor circuit remodeling is a clinically meaningful determinant of patient outcomes (46). Collectively, these studies demonstrate that neural tissue surrounding high-grade gliomas actively regulates tumor behavior through mechanisms shaped by preexisting network architecture and the transcriptional identity of individual tumor cell subtypes.

Tetzlaff et al. further demonstrated that glioblastoma rapidly integrates into diverse neural circuits across the cerebral cortex and engages in widespread functional communication with surrounding neurons (19). In this particular study, cholinergic neurons were identified as potentiators of glioblastoma invasion, with patient-specific and tumor-cell-state-dependent patterns of neural-tumor connectivity linked to enhanced invasiveness. Radiotherapy paradoxically increased surrounding neuronal activity, thereby enhancing neuron-tumor cell connectivity, whereas simultaneous inhibition of neuronal activity and radiotherapy improved therapeutic effects. These findings support the concept that neuron-to-glioma synapses contribute not only to tumor progression but also to therapeutic resistance. Therefore, neural circuit integration may be a key determinant of why glioblastoma behaves differently across patients, regions, and therapeutic contexts.

Emerging evidence suggests that this same neurobiological axis can also shape immune phenotype. Guo et al. reported that hypoxia drove glioma stem cells to release excess glutamate, activating local neurons and promoting microglial M2 polarization through neuron-derived exosomal signaling linked to RNA m6A demethylation (17). In the broader glioblastoma literature, related myeloid programs have been associated with diminished cytotoxic T-cell activity and impaired antigen presentation (49,50). In a separate study, Guo et al. showed that neuronal activity promoted glioma progression by inducing proneural-to-mesenchymal transition in glioma stem cells, a phenotype associated with greater aggressiveness and treatment resistance (51). From this perspective, the limited efficacy of current immunotherapy may partially reflect deployment into a niche that has already been biologically preconditioned against durable antitumor immunity. These observations suggest that the challenge may extend beyond insufficient immune activation to include upstream neuro-immune regulatory processes, in which the local brain environment may bias macrophage state, reinforce T-cell dysfunction, and constrain therapeutic responsiveness before treatment even begins.

### 4.3 Spatial heterogeneity

The second major challenge is that neuro-immunologic regulation is unlikely to be uniform across tumor-bearing regions. Adult-type diffuse gliomas are molecularly heterogeneous diseases, and glioblastoma is characterized by recurrent alterations affecting EGFR, PTEN, TP53, cell-cycle control, chromosomal stability, and telomere maintenance (3,4,6,52,53). These alterations coexist within tumors that are genetically, transcriptionally, immunologically, and spatially diverse (3,4,6,18,52,53). Such heterogeneity is clinically relevant because it increases the likelihood of antigen escape and allows for regional differences in treatment sensitivity, thereby reinforcing the concept that distinct tumor territories may exist throughout the brain (52,53).

Beyond intratumor spatial heterogeneity, inter-patient molecular diversity further complicates the neuro-immune landscape. Glioblastoma molecular subtypes–including mesenchymal, proneural, or classical transcriptional profiles–are associated with distinct immune compositions and interact with separate neuronal signaling pathways (3,4,6,52,53). Recent studies have shown that the mesenchymal subtype is largely enriched with immunosuppressive and proinflammatory genes, whereas the proneural and classical subtypes have demonstrated significantly lower levels of lymphocytic infiltration, which may partly account for differential immunotherapeutic responsiveness (54). This interconnection between inter-tumor molecular subtype and intratumor neuronal activity likely contributes to the variation in patient outcomes.

A spatial transcriptomic study integrating paired single-nuclei RNA sequencing and spatial transcriptomics data from three human glioblastoma patients reveals that glioblastoma is organized into distinct microanatomical niches with spatially segregated tumor cell states and markedly heterogeneous immune and metabolic composition (18). Liu et al. identified four transcriptionally defined tumor cell states—neural progenitor-like, oligodendrocyte progenitor-like (OPC-like), astrocyte-like (AC-like), and mesenchymal-like—and demonstrated that these states do no intermingle randomly but instead preferentially localize to distinct spatial regions. OPC-like tumor cells, for example, were consistently enriched near the tumor vasculature across samples, potentially reflecting higher oxygen requirements. Microglia and macrophages were most abundant in the necrotic, perinecrotic, and vessel-adjacent regions, and differentially expressed gene analysis identified upregulation of hypoxia-response and glycolytic genes—including ENO2, HILPDA, and CHI3L1—as the dominant transcriptional signature of the palisading necrotic niche. Functionally speaking, perinecrotic regions were substantially more immunosuppressive than the broader glioblastoma microenvironment, while perivascular regions displayed a comparatively pro-inflammatory signature enriched in oxidative phosphorylation pathways. These findings demonstrate that not all tumor territories are biologically equivalent with respect to immune response.

Recent work has added a neural dimension to this spatial model. Nejo et al. showed that high-grade glioma regions with enhanced neuronal activity exhibit regional immunosuppression characterized by distinct immune cell composition and enrichment of anti-inflammatory TAMs (20). Highly connected regions demonstrated suppression of interferon-driven responses as well as TNF/NFκB-related programs, while knockout mutations in tumor-associated synergistic signaling molecules (i.e., Thrombospondin-1) reduced glutamatergic hyperexcitability and altered immune architecture (20). These findings indicate that spatial heterogeneity in high-grade gliomas may partly reflect regional differences in neuronal activity, which consequently influence where immunosuppressive programs are most strongly expressed. If different tumor regions exhibit distinct neural and immune states, uniform therapies may therefore produce variable regional responses. In this framework, glioblastoma and other high-grade gliomas are better understood as patchworks of local neuro-immune ecosystems distributed across functional brain circuits, where spatial differences in neuronal signaling help explain regional variability in immune permissiveness and treatment outcomes.

### 4.4 Limitations and unresolved questions

Several limitations of the current evidence should be acknowledged. Much of the evidence linking neuronal activity to immune modulation in high-grade gliomas is derived from preclinical models. The extent to which these mechanisms function in human glioblastoma remains incompletely established. For example, while Guo et al. identified that hypoxia-driven neuronal activity promotes M2 polarization, the generalizability of this pathway has not been fully established (17). The causal directionality of neuron-glioma immune interactions remains an area of further research—whether the neuronal activity directly drives immunosuppression or whether tumor-integrated circuit remodeling indirectly shapes immune phenotype. Although current transcriptional studies reveal marked regional heterogeneity, this data is limited by the unpredictable nature of high-grade gliomas and may not yet capture the full extent or complexity of neuro-immune interactions across diverse patient populations and molecular subtypes. These limitations highlight the need for multidisciplinary and multimodal studies integrating immune phenotype, electrophysiology, and spatial transcriptomics in translating to human research.

## 5 Discussion

The emerging view of glioblastoma as a neuro-immunologic network challenges the traditional tumor-centric paradigm that has guided both surgical and systemic therapies. Glioblastoma exists within a dynamic ecosystem composed of tumor cells, immune populations, vascular structures, stromal elements, and neuronal circuits. Interactions among these components collectively contribute to tumor progression, immune evasion, and therapeutic resistance. Consequently, strategies directed solely toward enhancing antitumor immunity may be insufficient if the underlying neural, metabolic, and microenvironmental mechanisms that sustain immunosuppression remain intact.

### 5.1 Neuro-immune platform and therapeutic implications

The neuro-immune framework has direct implications for therapeutic design. First and foremost, targeting neuronal activity as an upstream regulator of immune suppression offers a mechanistically distinct avenue to reprogram the TME—one that differs fundamentally from approaches directed solely at downstream immune activation. The neuron-glioma signaling pathway outlined earlier collectively define a set of upstream intervention points that, if disrupted, could reduce the immunosuppressive baseline of the TME before immune effectors are deployed. A particularly informative example is the paradoxical observation that radiotherapy increases surrounding neuronal activity and neuron-tumor connectivity, which may partially explain early treatment resistance and argues for the concurrent disruption of neuronal inputs alongside standard chemoradiation (19).

Second, the neuro-immune axis represents a source of biomarkers for patient stratification. Intraoperative electrocorticography and connectome mapping can characterize the degree of tumor-neural circuit integration, as demonstrated by Krishna et al. (46), providing a clinically accessible metric that may predict immunotherapeutic responsiveness and overall survival. Similarly, molecular subtype classification—particularly identification of proneural-to-mesenchymal transition status—may inform which patients are most likely to benefit from approaches targeting both neural signaling and immune checkpoint inhibitors (54). Future biomarker development could incorporate spatial transcriptomic profiling of immune-neural niche composition alongside conventional tumor genomic characterization.

Third, this framework shapes how translational priorities should be ordered. Immunotherapy trials have largely enrolled molecularly and spatially heterogenous patient populations without accounting for neuro-immune landscape composition, which may partly explain variable clinical outcomes. The surgical and local therapeutic strategies discussed in the following sections represent an opportunity to incorporate neuro-immune biomarker endpoints—such as network integration scores, molecular subtype, and spatial immune-niche profiling—into trial design. It is important to note that most mechanistic evidence linking neuronal activity to immune suppression remains preclinical, and the degree to which these interactions are primary drivers of immune failure in human glioblastoma, as opposed to contributing factors among several parallel mechanisms, remains to be established through prospective human studies.

### 5.2 Implications for surgical management and the perioperative immune window

Within a network-centric framework, surgical resection can alter tumor burden, tissue architecture, antigen exposure, local immune traffic, and the timing of downstream therapy (55). Surgical resection not only reduces suppressive mass effect but can also influence cytokine gradients and transiently disrupt established neuron-glioma circuits, thereby creating a perioperative immunological window (55–58). Neoadjuvant immunotherapy studies convey the importance of timing; in recurrent glioblastoma, PD-1 blockade introduced before resection was associated with intratumoral immune activation, enhanced interferon-related signaling, and clonal T-cell expansion (57,58). NLGN3 expression levels, electrophysiologic activity, and spatial immune compositions may therefore serve as potential biomarkers that could inform patient stratification and perioperative therapeutic target selection.

### 5.3 Rethinking local therapy: from cytotoxicity to immunomodulation

Historically, carmustine placement has represented a tumor-centric approach to eliminate residual intracavitary cells with local chemotherapy (59). However, a network-focused shift proposes that surgical intervention can reprogram the local TME to prevent recurrence. Rising preclinical literature demonstrates that resection-cavity scaffolds, hydrogels, and local delivery platforms can postoperatively recruit lymphocytes, reprogram macrophages, and activate other immune pathways (60–63). Hydrogels are particularly advantageous in the postoperative setting as local immunotherapeutic platforms within the resection cavity; for example, Zhang et al. implemented an intracavitary, injectable hydrogel that functioned as a drug reservoir for CXCL10 and an immune nanoregulator, sustaining *in vivo* release and maintaining T-cell infiltration (62). These approaches differ from traditional designs of irrigating the cavity with chemotherapy, as they aim to reshape the local immune environment that determines early recurrence.

Local therapy is relevant because the resection cavity represents a clinically accessible interface between residual tumor cells, disrupted tissue architecture, and the postoperative immune microenvironment. Local platform deliveries such as hydrogels, scaffolds, and convection-enhanced delivery may provide a means to modulate immune-cell recruitment, macrophage polarization, cytokine signaling, and regional therapeutic exposure. Expectantly, local immunomodulatory therapy provides a practical neurosurgical route for applying preclinical neuro-immune mechanisms to the clinical management of glioblastoma.

### 5.4 The importance of spatial heterogeneity in therapeutic design

A challenge that arises from the previously mentioned designs is that tumor regions are not biologically equivalent with respect to variable neuronal connectivity, perinecrotic biology, and local immune composition (12,20). This raises the possibility that resection strategy, focused ultrasound, or convection-enhanced delivery could be tailored to region-specific neuro-immune features rather than uniform application (64–66). To that effect, maximal resection alone may be insufficient; rather, the goal is to identify and preferentially target the residual niches that are most likely to drive tumor recurrence. The implication is that, in this manner, surgery alone, even with gross total resection, is unlikely to fully address the challenges of glioblastoma and other high-grade gliomas. Rather, the intraoperative timing may represent a critical opportunity to intervene within the neuro-immune network. From a neuro-immune perspective, these platforms and early-phase clinical efforts, such as focused ultrasound and neoadjuvant checkpoint inhibitor trials, represent initial steps toward integrating microenvironmental modulation into therapeutic design.

### 5.5 Future directions and conclusions

Glioblastoma, IDH-wildtype, CNS WHO grade 4 is increasingly understood as a neuro-immunologic network in which tumor cells, immune populations, vascular structures, stromal components, and neuronal circuits interact to drive tumor progression and treatment resistance. Emerging evidence suggests that NLGN3-mediated signaling, neuron-glioma synaptic transmission, hypoxia-driven immune remodeling, and spatially restricted immune landscapes are interconnected processes that collectively shape tumor behavior and therapeutic outcomes.

Current immunotherapeutic strategies have shown limited efficacy in glioblastoma, partly because they primarily target downstream immune activation without fully addressing the structural, metabolic, and neural signaling pathways that sustain the immunosuppressive microenvironment. Within this framework, future treatment will likely require integrated approaches that combine spatially informed surgery, local immunomodulation, and targeted disruption of neuron-tumor interactions. Future clinical trial designs may benefit from integrating spatial and neural-circuit biomarkers to identify which patients are most likely to respond to neuro-immune targeted interventions.

## References

[1] Kanderi T Munakomi S Gupta V . Glioblastoma multiforme. In: Statpearls. Treasure Island, FL: StatPearls Publishing (2024). 32644380

[2] Lan Z Li X Zhang X . Glioblastoma: an update in pathology, molecular mechanisms and biomarkers. Int J Mol Sci (2024) 25(5):3040. 10.3390/ijms25053040 38474286 PMC10931698

[3] Berger TR Wen PY Lang-Orsini M Chukwueke UN . World Health Organization 2021 classification of central nervous system tumors and implications for therapy for adult-type gliomas: a review. JAMA Oncol (2022) 8(10):1493–501. 10.1001/jamaoncol.2022.2844 36006639

[4] Horbinski C Solomon DA Lukas RV Packer RJ Brastianos P Wen PY Molecular testing for the World Health Organization classification of central nervous system tumors: a review. JAMA Oncol (2025) 11(3):317–28. 10.1001/jamaoncol.2024.5506 39724142 PMC12344474

[5] Antonelli M Poliani PL . Adult type diffuse gliomas in the new 2021 WHO classification. Pathologica (2022) 114(6):397–409. 10.32074/1591-951X-823 36534419 PMC9763975

[6] van den Bent MJ Geurts M French PJ Smits M Capper D Bromberg JEC Primary brain tumours in adults. Lancet (2023) 402(10412):1564–79. 10.1016/S0140-6736(23)01054-1 37738997

[7] Fernandes C Costa A Osório L Lago RC Linhares P Carvalho B In: Current Standards of Care in Glioblastoma Therapy. Brisbane (AU): Codon Publications (2017). 10.15586/codon.glioblastoma.2017.ch11 29251860

[8] Stupp R Taillibert S Kanner A Read W Steinberg D Lhermitte B Effect of tumor-treating fields plus maintenance temozolomide vs maintenance temozolomide alone on survival in patients with glioblastoma: a randomized clinical trial. JAMA (2017) 318(23):2306–16. 10.1001/jama.2017.18718 29260225 PMC5820703

[9] Stupp R Mason WP van den Bent MJ Weller M Fisher B Taphoorn MJ Radiotherapy plus concomitant and adjuvant temozolomide for glioblastoma. N Engl J Med (2005) 352(10):987–96. 10.1056/NEJMoa043330 15758009

[10] Rocha Pinheiro SL Lemos FFB Marques HS Silva Luz M de Oliveira Silva LG Faria Souza Mendes Dos Santos C Immunotherapy in glioblastoma treatment: current state and future prospects. World J Clin Oncol (2023) 14(4):138–59. 10.5306/wjco.v14.i4.138 37124134 PMC10134201

[11] Zhang X Zhao L Zhang H Zhang Y Ju H Wang X The immunosuppressive microenvironment and immunotherapy in human glioblastoma. Front Immunol (2022) 13:1003651. 10.3389/fimmu.2022.1003651 36466873 PMC9712217

[12] Liu JH Horiachok M Guru S Maire CL . Unraveling glioblastoma heterogeneity: advancing immunological insights and therapeutic innovations. Brain Sci (2025) 15(8):833. 10.3390/brainsci15080833 40867165 PMC12384581

[13] Venkatesh HS Johung TB Caretti V Noll A Tang Y Nagaraja S Neuronal activity promotes glioma growth through neuroligin-3 secretion. Cell (2015) 161(4):803–16. 10.1016/j.cell.2015.04.012 25913192 PMC4447122

[14] Venkatesh HS Tam LT Woo PJ Lennon J Nagaraja S Gillespie SM Targeting neuronal activity-regulated neuroligin-3 dependency in high-grade glioma. Nature (2017) 549(7673):533–7. 10.1038/nature24014 28959975 PMC5891832

[15] Venkataramani V Tanev DI Strahle C Studier-Fischer A Fankhauser L Kessler T Glutamatergic synaptic input to glioma cells drives brain tumour progression. Nature (2019) 573(7775):532–8. 10.1038/s41586-019-1564-x 31534219

[16] Venkataramani V Yang Y Schubert MC Reyhan E Tetzlaff SK Wißmann N Glioblastoma hijacks neuronal mechanisms for brain invasion. Cell (2022) 185(16):2899–917.e31. 10.1016/j.cell.2022.06.054 35914528

[17] Guo X Qiu W Li B Qi Y Wang S Zhao R Hypoxia-induced neuronal activity in glioma patients polarizes microglia by potentiating RNA m6A demethylation. Clin Cancer Res (2024) 30(6):1160–74. 10.1158/1078-0432.CCR-23-0430 37855702

[18] Liu M Ji Z Jain V Smith VL Hocke E Patel AP Spatial transcriptomics reveals segregation of tumor cell states in glioblastoma and marked immunosuppression within the perinecrotic niche. Acta Neuropathol Commun (2024) 12(1):64. 10.1186/s40478-024-01769-0 38650010 PMC11036705

[19] Tetzlaff SK Reyhan E Layer N Bengtson CP Heuer A Schroers J Characterizing and targeting glioblastoma neuron-tumor networks with retrograde tracing. Cell (2025) 188(2):390–411.e36. 10.1016/j.cell.2024.11.002 39644898

[20] Nejo T Krishna S Yamamichi A Lakshmanachetty S Jimenez C Lee KY Glioma-neuronal circuit remodeling induces regional immunosuppression. Nat Commun (2025) 16(1):4770. 10.1038/s41467-025-60074-z 40404658 PMC12098748

[21] Xu L Chen S Fu Y Zhou T Yu J Li J Neuro-immune-tumor axis in gliomas: a review of mechanisms, models, and translational opportunities. Front Immunol (2025) 16:1682322. 10.3389/fimmu.2025.1682322 41132643 PMC12540510

[22] Dapash M Hou D Castro B Lee-Chang C Lesniak MS . The interplay between glioblastoma and its microenvironment. Cells (2021) 10(9):2257. 10.3390/cells10092257 34571905 PMC8469987

[23] Mohiuddin E Wakimoto H . Extracellular matrix in glioblastoma: opportunities for emerging therapeutic approaches. Am J Cancer Res (2021) 11(8):3742–54. 34522446 PMC8414390

[24] Pantazopoulou V Jeannot P Rosberg R Berg TJ Pietras A . Hypoxia-induced reactivity of tumor-associated astrocytes affects glioma cell properties. Cells (2021) 10(3):613. 10.3390/cells10030613 33802060 PMC7999295

[25] Talukdar S Pradhan AK Bhoopathi P Shen XN August LA Windle JJ MDA-9/Syntenin regulates protective autophagy in anoikis-resistant glioma stem cells. Proc Natl Acad Sci USA (2018) 115(22):5768–73. 10.1073/pnas.1721650115 29760085 PMC5984507

[26] Abbas S Singh SK Saxena AK Tiwari S Sharma LK Tiwari M . Role of autophagy in regulation of glioma stem cells population during therapeutic stress. J Stem Cells Regen. Med. (2020) 16(2):80–9. 10.46582/jsrm.1602012 33414584 PMC7772813

[27] Shi T Zhu J Zhang X Mao X . The role of hypoxia and cancer stem cells in development of glioblastoma. Cancers (2023) 15(9):2613. 10.3390/cancers15092613 37174078 PMC10177528

[28] Khaddour K Johanns TM Ansstas G . The landscape of novel therapeutics and challenges in glioblastoma multiforme: contemporary state and future directions. Pharmaceuticals (2020) 13(11):389. 10.3390/ph13110389 33202642 PMC7696377

[29] Hu J Li X Yang L Li H . Hypoxia, a key factor in the immune microenvironment. Biomed Pharmacother (2022) 151:113068. 10.1016/j.biopha.2022.113068 35676780

[30] Aitchison EE Dimesa AM Shoari A . Matrix metalloproteinases in glioma: drivers of invasion and therapeutic targets. BioTech (2025) 14(2):28. 10.3390/biotech14020028 40265458 PMC12015896

[31] Bajetto A Thellung S Dellacasagrande I Pagano A Barbieri F Florio T . Cross talk between mesenchymal and glioblastoma stem cells: communication beyond controversies. Stem Cells Transl. Med. (2020) 9(11):1310–30. 10.1002/sctm.20-0161 32543030 PMC7581451

[32] Kim SM Lim EJ Yoo KC Zhao Y Kang JH Lim EJ Glioblastoma-educated mesenchymal stem-like cells promote glioblastoma infiltration *via* extracellular matrix remodelling in the tumour microenvironment. Clin Transl Med (2022) 12(8):e997. 10.1002/ctm2.997 35908277 PMC9339241

[33] Hossain A Gumin J Gao F Figueroa J Shinojima N Takezaki T Mesenchymal stem cells isolated from human gliomas increase proliferation and maintain stemness of glioma stem cells through the IL-6/gp130/STAT3 pathway. Stem Cells (2015) 33(8):2400–15. 10.1002/stem.2053 25966666 PMC4509942

[34] Lamano JB Lamano JB Li YD DiDomenico JD Choy W Veliceasa D Glioblastoma-derived IL6 induces immunosuppressive peripheral myeloid cell PD-L1 and promotes tumor growth. Clin Cancer Res (2019) 25(12):3643–57. 10.1158/1078-0432.CCR-18-2402 30824583 PMC6571046

[35] Frantz C Stewart KM Weaver VM . The extracellular matrix at a glance. J Cell Sci. (2010) 123(24):4195–200. 10.1242/jcs.023820 21123617 PMC2995612

[36] Silva-Pavez E Urra H . LOX rises as a potential survival biomarker: a commentary on identification of LOX as a candidate prognostic biomarker in glioblastoma multiforme. Liu Al Transl Oncol (2024) 44:101777. 10.1016/j.tranon.2023.101777 38641375 PMC11391031

[37] Arvanitis CD Ferraro GB Jain RK . The blood-brain barrier and blood-tumour barrier in brain tumours and metastases. Nat Rev Cancer (2020) 20(1):26–41. 10.1038/s41568-019-0205-x 31601988 PMC8246629

[38] Dotiwala AK McCausland C Samra NS . Anatomy, head and neck: Blood-brain barrier. In: Statpearls. Treasure Island, FL: StatPearls Publishing (2023). 30137840

[39] Abikenari M Sjoholm MA Liu J Nageeb G Ha JH Wu J Molecular and biophysical remodeling of the blood-brain barrier in glioblastoma: mechanistic drivers of tumor-neurovascular crosstalk. Front Phys (2025) 13:1723329. 10.3389/fphy.2025.1723329

[40] Yang J Yan J Liu B . Targeting VEGF/VEGFR to modulate antitumor immunity. Front Immunol (2018) 9:978. 10.3389/fimmu.2018.00978 29774034 PMC5943566

[41] Sarfraz Z Maharaj A Venur VA Lathia JD Odia Y Ahluwalia MS . Immunotherapy in glioblastoma: an overview of current status. Clin Pharmacol (2025) 17:185–209. 10.2147/CPAA.S497903 40735070 PMC12305673

[42] Sadelain M Brentjens R Rivière I . The basic principles of chimeric antigen receptor design. Cancer Discov (2013) 3(4):388–98. 10.1158/2159-8290.CD-12-0548 23550147 PMC3667586

[43] Iranzo P Callejo A Assaf JD Molina G Lopez DE Garcia-Illescas D Overview of checkpoint inhibitors mechanism of action: role of immune-related adverse events and their treatment on progression of underlying cancer. Front Med (2022) 9:875974. 10.3389/fmed.2022.875974 PMC9189307 35707528

[44] Ghouzlani A Kandoussi S Tall M Reddy KP Rafii S Badou A . Immune checkpoint inhibitors in human glioma microenvironment. Front Immunol (2021) 12:679425. 10.3389/fimmu.2021.679425 34305910 PMC8301219

[45] Del BG Del Bufalo F Pinacchio C Carai A Quintarelli C De Angelis B The peculiar challenge of bringing CAR-T cells into the brain: perspectives in the clinical application to the treatment of pediatric central nervous system tumors. Front Immunol (2023) 14:1142597. 10.3389/fimmu.2023.1142597 37025994 PMC10072260

[46] Krishna S Choudhury A Keough MB Seo K Ni L Kakaizada S Glioblastoma remodelling of human neural circuits decreases survival. Nature (2023) 617(7961):599–607. 10.1038/s41586-023-06036-1 37138086 PMC10191851

[47] Greenwald AC Darnell NG Hoefflin R Simkin D Mount CW Gonzalez Castro LN Integrative spatial analysis reveals a multi-layered organization of glioblastoma. Cell (2024) 187(10):2485–501.e26. 10.1016/j.cell.2024.03.029 38653236 PMC11088502

[48] Huang-Hobbs E Cheng YT Ko Y Luna-Figueroa E Lozzi B Taylor KR Remote neuronal activity drives glioma progression through SEMA4F. Nature (2023) 619(7971):844–50. 10.1038/s41586-023-06267-2 37380778 PMC10840127

[49] Ravi VM Neidert N Will P Joseph K Maier JP Kückelhaus J T-cell dysfunction in the glioblastoma microenvironment is mediated by myeloid cells releasing interleukin-10. Nat Commun (2022) 13(1):925. 10.1038/s41467-022-28523-1 35177622 PMC8854421

[50] Schartner JM Hagar AR Van Handel M Zhang L Nadkarni N Badie B . Impaired capacity for upregulation of MHC class II in tumor-associated microglia. Glia (2005) 51(4):279–85. 10.1002/glia.20201 15818597

[51] Guo X Qiu W Wang C Zhao R Wang S Qi Y Neuronal activity promotes glioma progression by inducing proneural-to-mesenchymal transition in glioma stem cells. Cancer Res (2024) 84(3):372–87. 10.1158/0008-5472.CAN-23-0609 37963207

[52] Crespo I Vital AL Gonzalez-Tablas M Otero A Lopes MC de Oliveira C Molecular and genomic alterations in glioblastoma multiforme. Am J Pathol (2015) 185(7):1820–33. 10.1016/j.ajpath.2015.02.023 25976245

[53] Prakash P Trippett J Ehsan C Namkung J Lad M Aghi MK . Tumor microenvironment shapes the spatial organization of glioblastoma cell states. Neuro Oncol (2026) 28(3):585–96. 10.1093/neuonc/noag003 41520155 PMC13070500

[54] Kaffes I Szulzewsky F Chen Z Herting CJ Gabanic B Velázquez Vega JE Human mesenchymal glioblastomas are characterized by an increased immune cell presence compared to proneural and classical tumors. Oncoimmunology (2019) 8(11):e1655360. 10.1080/2162402X.2019.1655360 31646100 PMC6791439

[55] Duerinck J Tuyaerts S Movahedi K Neyns B . Overcoming the immune suppressive nature of glioblastoma by leveraging the surgical intervention-current status and future perspectives. Front Immunol (2023) 14:1183641. 10.3389/fimmu.2023.1183641 37275902 PMC10237336

[56] Choi SH Stuckey DW Pignatta S Reinshagen C Khalsa JK Roozendaal N Tumor resection recruits effector T cells and boosts therapeutic efficacy of encapsulated stem cells expressing IFNβ in glioblastomas. Clin Cancer Res (2017) 23(22):7047–58. 10.1158/1078-0432.CCR-17-0077 28912136 PMC6818096

[57] Cloughesy TF Mochizuki AY Orpilla JR Hugo W Lee AH Davidson TB Neoadjuvant anti-PD-1 immunotherapy promotes a survival benefit with intratumoral and systemic immune responses in recurrent glioblastoma. Nat Med (2019) 25(3):477–86. 10.1038/s41591-018-0337-7 30742122 PMC6408961

[58] Schalper KA Rodriguez-Ruiz ME Diez-Valle R López-Janeiro A Porciuncula A Idoate MA Neoadjuvant nivolumab modifies the tumor immune microenvironment in resectable glioblastoma. Nat Med (2019) 25(3):470–6. 10.1038/s41591-018-0339-5 30742120

[59] Westphal M Lamszus K Hilt D . Intracavitary chemotherapy for glioblastoma: present status and future directions. Acta Neurochir Suppl (2003) 88:61–7. 10.1007/978-3-7091-6090-9_11 14531563

[60] Sabahi M Salehipour A Bazl MSY Rezaei N Mansouri A Borghei-Razavi H . Local immunotherapy of glioblastoma: a comprehensive review of the concept. J Neuroimmunol (2023) 381:578146. 10.1016/j.jneuroim.2023.578146 37451079

[61] Graham-Gurysh EG Woodring RN Simpson SR Mendell SE Lukesh NR Pena ES Post-resection delivery of a TLR7/8 agonist from a biodegradable scaffold achieves immune-mediated glioblastoma clearance and protection against tumor challenge in mice. Nat Commun (2025) 16(1):8603. 10.1038/s41467-025-63692-9 41022761 PMC12479796

[62] Zhang J Chen C Li A Zhang X Shen Y Zhao Y Immunostimulant hydrogel for the inhibition of malignant glioma relapse post-resection. Nat Nanotechnol (2021) 16(5):538–48. 10.1038/s41565-020-00843-7 33526838

[63] Zhu M Yang M Li R Hu X Chen J Xu L Local therapeutic platform prevents postsurgical GBM recurrence by diminishing GICs and reshaping immunosuppressive microenvironment. Nat Commun (2025) 17(1):636. 10.1038/s41467-025-67415-y 41413041 PMC12816023

[64] Uribe Cardenas R Laramee M Ray I Dahmane N Souweidane M Martin B . Influence of focused ultrasound on locoregional drug delivery to the brain: potential implications for brain tumor therapy. J Control Release (2023) 362:755–63. 10.1016/j.jconrel.2023.08.060 37659767

[65] Boroumand A Mehrarya M Ghanbarzadeh-Dagheyan A Ahmadian MT . Numerical and experimental evaluation of ultrasound-assisted convection enhanced delivery to transfer drugs into brain tumors. Sci Rep (2022) 12(1):19299. 10.1038/s41598-022-23429-w 36369259 PMC9652304

[66] Woodworth GF Anastasiadis P Ozair A Chabros J Bettegowda C Chen C Microbubble-enhanced transcranial focused ultrasound with temozolomide for patients with high-grade glioma (BT008NA): a multicentre, open-label, phase 1/2 trial. Lancet Oncol (2025) 26(12):1651–64. 10.1016/S1470-2045(25)00492-9 41308679 PMC12757104

